# An improved mathematical model for hypothetical oil reservoir for optimum oil recovery using magnetic nanomaterials

**DOI:** 10.1371/journal.pone.0328661

**Published:** 2025-08-13

**Authors:** Mudasar Zafar, Hamzah Sakidin, Abida Hussain, Ahmed Daabo, Farman Ullah, Rajasegeran Ramasamy, Roslinda Nazar, Mikhail Sheremet, Abdullah Al-Yaari, Iliyas Karim Khan

**Affiliations:** 1 School of Mathematics, Actuarial and Quantitative Studies (SOMAQS), Asia Pacific University of Technology and Innovation (APU), Bukit Jalil, Kuala Lumpur, Malaysia; 2 Department of Fundamental and Applied Sciences, Universiti Teknologi PETRONAS, Bandar Seri Iskandar, Perak, Malaysia; 3 Mining Engineering Department, College of Petroleum and Mining Engineering, The University of Mosul, Mosul, Iraq; 4 Department of Physics, University of Science and Technology, Bannu, Khyber Pakhtunkhwa, Pakistan; 5 Department of Mathematical Sciences, Faculty of Science & Technology, Universiti Kebangsaan Malaysia, UKM Bangi, Selangor, Malaysia; 6 Laboratory on Convective Heat and Mass Transfer, Tomsk State University, Tomsk, Russia; 7 Department of Mathematics, Faculty of Applied Science, Thamar University, Dhamar, Yemen; Dawood University of Engineering and Technology, PAKISTAN

## Abstract

In the oil and gas industry, enhanced oil recovery (EOR) strategies for unconventional reservoirs, characterized by complex geometries, differ significantly from those used in conventional reservoirs. This research focuses on the impact of 3D hexagonal prism geometries on EOR in hypothetical oil reservoirs using silicon dioxide (SiO₂) magnetic nanoparticles under liquid-phase flow conditions, a topic not extensively explored in existing literature. We developed an improved magnetohydrodynamic (MHD) mathematical models to simulate oil recovery processes in these geometries, using ANSYS Fluent for finite volume analysis. We developed an improved magnetohydrodynamic (MHD) model by incorporating magnetic field-induced pressure terms, nanoparticle transport losses, and a 3D hexagonal prism geometry that reflects complex reservoir behavior. These enhancements extend beyond traditional Darcy-based models by integrating magnetic permeability, viscosity alteration, and magnetic field-pore interactions. The model evaluates the impact of key reservoir parameters including porosity (ϕ = 0.1–0.4), injection flow rate (0.01–0.05 mL/min), and nanoparticle concentration (Ψ = 0.01–0.04), under different magnetic field configurations. Porosity and flow rate were also found to significantly influence recovery performance, highlighting the practical adaptability of the model for diverse reservoir conditions. Findings indicate that proximity of a magnetic field to cavity structures enhances oil recovery rates, with a significant 29.08% increase in recovery from nanoflooding compared to water flooding.Future research will extend this framework to study green, eco-friendly nanoparticles under elevated temperature and pressure, aiming to improve thermal stability, reduce environmental risks, and enhance recovery efficiency in more extreme reservoir conditions.

## 1. Introduction

Within the petroleum sector, two primary categories of oil reservoirs are recognized such as conventional and unconventional [1]. The oil recovery obtained from these reservoirs consists of three stages including primary, intermediate, and tertiary (EOR) recovery [2]. In unconventional oil extraction, primary recovery involves utilizing the natural pressure exerted by the oil and gas within the reservoir to obtain oil. Later, in the intermediate phase, external pressure is utilized to improve oil extraction. Finally, in the Enhanced Oil Recovery (EOR) phase, an artificial reservoir is established alongside the unconventional reservoirs to optimize oil extraction [3]. The design of various configurations within these artificial reservoirs is a crucial aspect in maximizing oil recovery. The exploration of unconventional reservoirs holds significance due to their substantial untapped resources [4]. Understanding the complex geology and extraction mechanisms of these reservoirs is vital for refining recovery strategies and meeting future energy demands [5,6].

Mathematical modeling plays a pivotal role in enhanced oil recovery as it enables researchers, technologist and industrialist to simulate and understand the complex fluid flow dynamics within the reservoir. This aids in devising optimal injection strategies and forecasting reservoir performance across diverse scenarios [7–16] refined the model to improve the precision of surface deposition estimation. Goldberg et al. [17] explored the efficacy of nanoparticle transport models in predicting flow in a saturated porous medium. The models developed by Salama et al. [18] utilized the flow equation, deposition term, remobilization, and obstacle-induced nanoparticle transfer to classify them. The authors suggested that the choice of modeling approach does not consistently impact nanoparticle prediction outcomes.

Ju and Fan [16] developed a mathematical model of nanoparticle transport in porous media based on several hypotheses, namely, (a) fluid flow is one-dimensional under isothermal conditions due to the incompressibility of rock and fluids; (b) the porous medium is homogeneous; (c) Darcy law governs the flow of water and oil in porous media, neglecting gravitational effects; (d) nanomaterials are discretized into n-sized sub-intervals; and (e) fluid viscosity and density remain constant. This model not only aids in predicting the permeability and porosity of the reservoir but also forecasts the amount of oil recoverable after nanofluid injection. After injecting nanoparticles into the reservoir, Researchers [19] developed the equations to determine changes in reservoir porosity and permeability. Implicit pressure-explicit saturation technique was employed to overcome issues with the model, effectively computing nanoparticle mobility in porous media. This model, an early and influential contribution, has been frequently cited by subsequent researchers in their efforts to comprehend nanoparticle movement in porous materials. It serves as a fundamental tool for understanding nanoparticle behavior in porous materials.

Cullen et al. [20], introduced a technique for estimating nanoparticle entrapment, employing the two-site model proposed by Zhang et al. [21] to compute the loss term. In [22,23] utilized the carbonate system model and validated its precision with experimental data, revealing the 8–10% increase in the recovery factor attributable to wettability changes compared to conventional water flooding for enhanced oil recovery (EOR). Precise determination of the nanoparticle loss term is critical for predicting nanoparticle migration in porous materials. Wettability of the core surface and well displacement were projected using numerical simulations based on the drag reduction model established by Chen et al. [24]. Abdelfatah [25] developed a mathematical model for nanofluid injection in heterogeneous porous media to identify optimal nanoparticle physical properties for EOR applications. El-Amin et al. [26] introduced several modifications for nanoparticle multiphase flow in porous media, with additional adaptations for CO_2_ sequestration by [27]. The equations were solved using a modified variant of the iterative IMPES approach, customized by the authors to enhance computational efficiency.

MHD (Magnetohydrodynamics) modeling provides insight into the interaction between magnetic fields and fluids within the reservoir. By manipulating fluid behavior with magnetic forces, displacement efficiency can be enhanced, resulting in greater oil recovery. Magnetic fields can modify the viscosity of reservoir fluids, rendering them more mobile and aiding their flow through porous rock formations. This alteration boosts sweep efficiency and ensures a higher proportion of oil is extracted [28]. Researchers suggest that the utilization of magnetic nanoparticles further enhances the rate of oil recovery [29–33].

In addition, magnetic nanoparticles have been shown to be effective in modifying the rock–fluid interface in tight and low-permeability reservoirs. Their small size enables better penetration into micro-pores, enhancing contact with trapped oil, while their surface activity promotes wettability alteration from oil-wet to water-wet conditions [34–36]. These mechanisms collectively improve sweep efficiency and are essential in heterogeneous reservoirs, as supported by [37,38].

Beyond their magnetically responsive behavior, nanoparticles play crucial standalone roles in EOR. Several studies have reported that nanoparticles such as SiO₂ and ZnO alter the wettability of reservoir rocks from oil-wet to water-wet, enhancing oil displacement efficiency [39–42]. Moreover, nanoparticles can reduce interfacial tension (IFT) between oil and water, improving capillary-driven recovery. In polymer flooding systems, nanoparticles help mitigate polymer adsorption on rock surfaces, preserving mobility control and chemical efficiency. These multi-functional mechanisms support the use of nanomaterials in non-polymeric and magnetic EOR strategies [43–46]. According to the preceding literature, our basic understanding of MHD modeling using magnetic nanoparticle assisted by EOR is extremely limited. We require a more in-depth theoretical understanding of how magnetic nanoparticles behave in oil reservoirs and under different physical environments. As a result, further models based on the investigation of external magnetic field source for nanoparticle flow in porous media are required. In this research we develop MHD mathematical model for 3D porous hexagonal prisms to predict the oil recovery using magnetic nanoparticles. According to this brief review the MHD mathematical model using 3D hexagonal prism is not studied.

## 2. Materials and methodology

**Fig 1:**
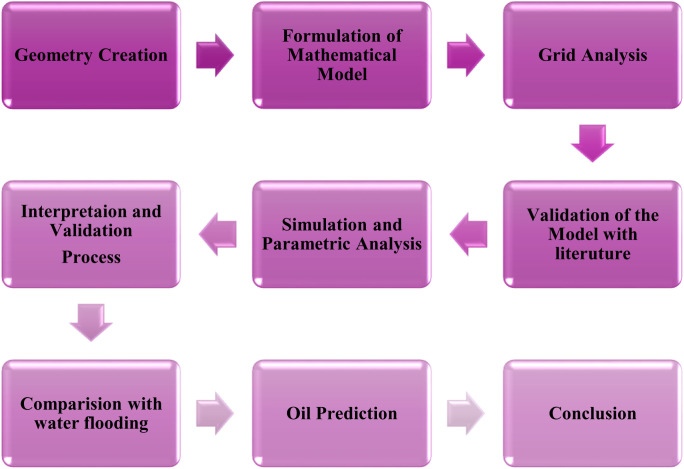
Flow of methodology.

In this section, we will explain the methodology of the research to predict oil recovery in 3D hexagonal prism cavities in hypothetical oil reservoir. The flow diagram of the methodology of this article is given in Fig 1. The present study is conducted using simulation-based analysis, employing a finite volume solver in ANSYS Fluent. It models the behavior of liquid-phase nanofluids within a porous reservoir structure. While the model does not represent a real-world field deployment, it is structured to reflect conditions encountered in laboratory-scale liquid-phase EOR studies. The hexagonal prism was selected as the simulation geometry due to its resemblance to natural pore structures in sedimentary formations. Unlike rectangular or spherical shapes, the hexagonal layout offers improved tessellation and reduced edge distortion, allowing for more accurate simulations of interstitial flow dynamics.

According to Fig 1, the methodology for our problem consists of the stages listed below.

- **Step 1**: In the first stage we will create or construct 3D hexagonal prism geometry using ANSYS Fluent software.
- **Step 2**: The second stage consists of the development of a two-phase mathematical model for our research problem involving externally applied magnetic fields for nanofluid injection with nanoparticles (NP). The general model comprises partial differential equations such as the Darcy equation, the saturation equation, and the nanoparticle concentration equation.
- **Step 3**: In the third step subsequently, mesh analysis will be conducted, and these equations will be discretized using the Finite Volume Method (FVM) solver.
- **Step 4**: In the fourth step, validation of our model with previously published experimental results is performed.
- **Step 5**: In the last step, We present and analyze the simulation results to evaluate oil recovery behavior under various parametric conditions to determine the oil recovery factor at different pore volumes using various parameters such as the external magnetic effect, gravitational effect, time impact on oil recovery, flooding behavior at different flow rates, different nanoparticle volume fractions, diverse nanoparticle effects on various porosity parameters, etc.

### 2.1. Construction of 3D prism cavity

**Fig 2:**
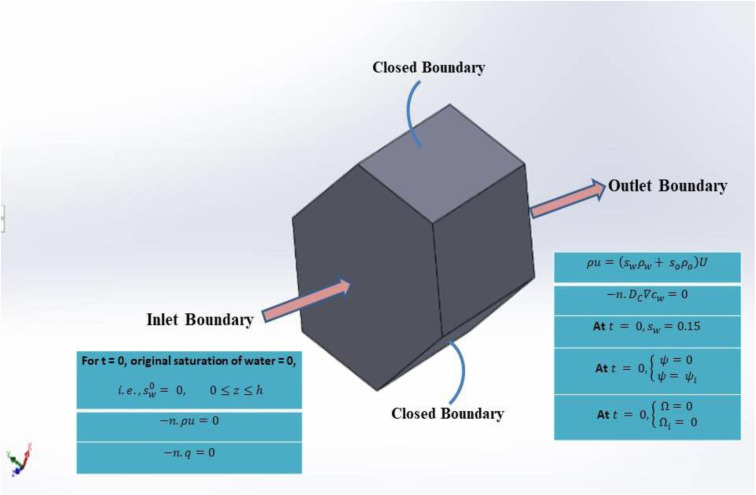
3D hexagonal prism cavity.

To construct geometry and to predict the optimal oil recovery in hypothetical oil reservoir we create the design of the 3D hexagonal prism in ANSYS software which is shown in Fig 2. The nanofluid consists of silicon dioxide (SiO₂) nanoparticles dispersed in water, chosen for their thermal stability and EOR efficiency.

The properties of the created 3D cavity are provided in Table 1.

**Table 1:** The parameters if the created 3D prism.

Parameters	Input Values
Largest Width	0.30 m
In radius	0.12 m
Circumference	0.15 m
Smallest width	0.25 m
Length of the side	0.14 m
Core Volume of prism	0.049 $m^{3}$
Inlet Boundary area of prism	0.45 $m^{2}$

Table 1 explains the values which were used in the construction of 3D prism design. It should be noted that during the flooding phenomenon the external perpendicular magnetic field was used for the prism geometry to investigate its effect on the oil recovery rate. During the nanoflooding phenomenon, the silica nanoparticles were used. Chemical properties of nanoparticles are shown in Table 2, while the reservoir parameters are presented in Table 3. The Rock properties are shown in Table 4.

**Table 2:** Nanoparticles chemical properties of nanoparticles in prism flooding [10].

Properties	Input Values
Density of the silica	2220 kgm^ − 3^
Specific heat of silica	745 J/Kg. K
Thermal conductivity of silica	36 W/m. K
Nanoparticles volume fraction	0.01
Diameter of silica	40 nm
Molecular mass of silica	60 g/mol

**Table 3:** Properties of reservoir in prism flooding.

Properties	Input Values
Density of the rock	2714 kgm^ − 3^
Diameter of the mesh	3 µm
Oil viscosity	1.15 × 10^2^ Pa. s
Water viscosity	10^ − 3^ Pa. s
Density of the oil	829 kgm^ − 3^
Density of the water	990 kgm^ − 3^

**Table 4:** Reservoir rock and fluid parameters.

Parameter	Value(Range)	Unit	Remarks
Porosity	0.1–0.4	–	Variable per scenario
Permeability	Assumed constant	$m^{2}$	Homogeneous medium
Relative Permeability	Modelded as function	–	From core assumptions
Oil Saturation	0.85(initially)	–	Initial saturation
Water Saturation	0.15(initially)	–	From boundary condition
Capillary Pressure	Based on Brooks-Corey Eq.	Pa	Varies with effective saturation

It is important to clarify that the nanoparticle volume fraction refers to the proportion of nanoparticles in the injected base fluid (water), not relative to the total volume of the reservoir or core. This ratio influences the thermophysical properties of the nanofluid, directly affecting its flow characteristics and oil displacement efficiency. The hexagonal prism configuration was chosen as a representative structure inspired by the natural tessellation found in porous sedimentary formations. This geometry allows more accurate simulation of interstitial flow and provides a compromise between computational tractability and physical relevance in pore-scale modeling.

### 2.2. Formulation of the model

The mathematical model consists of non-linear partial differential equation (PDEs) in the presence of the external magnetic field. The following hypotheses were used for the development of 3D hexagonal prism cavity to predict optimal oil recovery.

i The fluid flow inside the prism is one-dimensional.
ii The rock type is assumed to be pristine and transparent.
iii Fluid compression within the prism is negligible.
iv Darcy law governs the flooding process.
v Chemical process outcomes are disregarded.
vi Temperature remains constant within the cavity.
vii Nanofluid viscosity follows Newtonian behavior, and gravitational effects are not ignored.
viii Nanoparticles are exclusively present in water.
ix External magnetic forces act perpendicular to the prism.

The system of governing equations for developing the model for 3D hexagonal prism is as follows [11,43,46]. Our modeling framework incorporates several novel aspects compared to existing nanofluid EOR models. These include: (i) coupling magnetic forces with capillary and magnetostriction pressure terms, (ii) integration of nanoparticle transport loss through kinetic absorption/desorption models, and (iii) extension to a 3D hexagonal prism structure to better capture realistic pore-scale interactions. The assumptions adopted such as incompressible flow, constant temperature, and Newtonian behavior are consistent with prior validated studies and enable stable finite-volume simulations.


$$\frac{\partial {\varphi S}_{\delta }}{\partial t}+\;\nabla .\left(\frac{u_{\delta }}{\mu_{\delta }}\right)=0,\;where\;\delta =\;o,\;w\;and\;u=kK_{r\;\delta }[\frac{\partial p_{\;\delta }}{\partial z}-\;\rho g]$$ (1)

The continuity equation for the oil phase is defined in Eq. (2)


$$\frac{\partial {\varphi S}_{o}}{\partial t}+\;\nabla .\left(\frac{kK_{ro}}{\mu_{o}}[\frac{\partial p_{o}}{\partial z}-\;\rho g]\right)=0$$ (2)

It is important to note that the external magnetic field influences the water phase equation, and the velocity for both the water and hydrocarbon phases is defined by Eq. (3).


$$\frac{\partial {\varphi S}_{w}}{\partial t}+\;\nabla .\left(\frac{kK_{rw}}{\mu_{w}}[\frac{\partial p_{w}}{\partial z}-\;\rho g-F_{mag}]\right)=0$$ (3)

The values of $F_{mag}$ can be determined by using Eq. (4),


$$F_{mag}\;=\mu_{o}M\frac{\partial H}{\partial z}$$ (4)

In Eq. (4), $\mu_{o}$ denotes the permeability of the magnetic field, which can be computed using Eq. (5), and the values of *M* can be determined using Eq. (6).


$$\mu_{o}\;=\frac{B}{H}$$ (5)

$$M=\;a_{1\;}\tan^{-1}\left(b_{1}H\right)$$ (6)

As shown in Eq. (6), the values of *a*_1_ and *b*_1_ vary depending on the magnetic nanoparticles utilized. The parameter *a*_1_ ranges between 10^–5^ and 10^–6^, while the parameter *b*_1_ falls within the range of 10^–5^ and 10^–6^. Employing Eq. (7), we can calculate the magnetic field strength *H* as follows.


$$H_{z}=\;\frac{B_{r}}{\pi \mu_{0}}(\;\tan^{-1}\frac{ab}{z\sqrt{a^{2}+b^{2}+z^{2}}}-\tan^{-1}\frac{ab}{(z+l)\sqrt{a^{2}+b^{2}+{\left(z+l\right)}^{2}}}$$ (7)

The residual magnetization, represented by *B*_*r*_ in Eq. (7), is determined by the distance *l* between the magnetic poles. Density and viscosity, that are essential for solving the Darcy equations, can be derived using the following equations [29].


$$\rho =\;s_{w\;}\rho_{w}+s_{o}\rho_{o}$$ (8)

$$\frac{1}{\mu }\;=\;s_{water\;}\frac{k_{rw}}{\mu_{rw}}+s_{oil\;}\frac{k_{r0}}{\mu_{o}}$$ (9)

In the current model fluid saturation specifies as


$$s_{w\;}+s_{o}=1$$ (10)

Additionally, both the wetting and non-wetting phases contribute to a total velocity of zero


$$u_{w\;}+u_{o}=0$$ (11)

In the current model pressure is defined in Eq. (12) as follows


$$p_{o\;}-p_{w}=p_{c}$$ (12)

Both magnetic and nonmagnetic phases coexist in this model, with an additional pressure term presented in the magnetic phase due to the influence of an external magnetic field. The resulting equation for pressure is


$$p_{w}^{*}=\;p_{w\;}+{(p}_{m}+p_{s}+p_{n})$$ (13)

In Eq. (13), *p*_*m*_ represents the magnetic pressure, *p*_*s*_ defines magnetostriction, and *p*_*n*_ defines magnetic normal pressure. In this study, we assume that *p*_*n*_ is zero. Now, Eq. (13) can be written as


$$p_{c}^{*}=\;p_{c\;}+{(p}_{m}+p_{s})$$ (14)

The values of *p*_*m*_ and *p*_*s*_ can be evaluated using Eqs. (15) and (16)


$$p_{s}=\;\mu_{0}\int V\frac{\partial M}{\partial V}\;dH$$ (15)

$$p_{m}=\;\mu_{0}\int M\;dH$$ (16)

The relation for *V* is defined as,


$$V=\;\frac{-1}{\rho^{2}}\;d\rho$$ (17)

$$p_{s}=\;-\mu_{0}\int \rho \frac{\partial M}{\partial \rho }\;dH$$ (18)

By using Chain Rule $\frac{\partial M}{\partial \rho }$ can be define as,


$$\frac{\partial M}{\partial \rho }=\;\frac{\partial M}{\partial a_{1}}\;.\;\frac{\partial a_{1}}{\partial \rho }+\;\frac{\partial M}{\partial b_{1}}\;.\;\frac{\partial b_{1}}{\partial \rho }+\frac{\partial M}{\partial H}.\frac{\partial H}{\partial \rho }$$ (19)

It is also assuming that *a*_1,0_ and *b*_1,0_ is zero due to nonmagnetic phase that


$$\;\frac{\partial a_{1}}{\partial \rho }\;\approx \;\frac{a_{1}-a_{1,o}}{\rho_{w}-\;\rho_{o}}\;\approx \;\frac{a_{1}}{\rho_{w}}$$ (20)

$$\;\frac{\partial b_{1}}{\partial \rho }\;\approx \;\frac{b_{1}-b_{1,o}}{\rho_{w}-\;\rho_{o}}\;\approx \;\frac{b_{1}}{\rho_{w}}$$ (21)

$$p_{s}=\frac{\rho_{wa_{1}}}{\rho_{w}-\;\rho_{o}}\mu_{0}H\tan^{-1}\left(b_{1}H\right)$$ (22)

$$p_{m}=a_{1}(H\tan^{-1}\left(b_{1}H\right)-\;\frac{1}{2b_{1}}\;ln\;(b_{1}^{2}H^{2}+1)$$ (23)

Now putting Eqs. (9), (18)–(21) in Eq. (23) we can evaluate the pressure term as


$$\frac{\partial p_{w}^{*}}{\partial z}=-f_{w}\frac{\partial p_{c}^{*}}{\partial z}+{(f}_{w\;}\rho_{w}+f_{o}\rho_{o})g+f_{w\;}\mu_{0}M\frac{\partial H}{\partial Z}$$ (24)

$$u_{w}=k\gamma_{w}f_{o}(\frac{\partial p_{c}^{*}}{\partial z}-\nabla \rho g+f_{w}\mu_{0}M\frac{\partial H}{\partial Z}\;)$$ (25)

Finally, the saturation equation of the water is defined as


$$\frac{\partial {\varphi S}_{w}}{\partial t}+\;\nabla .\left(k\gamma_{w}f_{o}(\frac{\partial p_{c}^{*}}{\partial z}-\nabla \rho g+f_{w}\mu_{0}M\frac{\partial H}{\partial Z}\;)\right)=0$$ (26)

In above equations, γ_*w*_ and γ_*o*_ are the mobility ratio of the oil and water phase, respectively, whereas *f*_*w*_ and *f*_*o*_ are the flow fractions of water and oil phases, respectively.

Capillary pressure is calculated using the logarithmic model and the mathematical equations of Brooks Corey.


$$p_{c}=\;-\;B_{C}\;\times \log\left(\;S_{e}\right)$$ (27)

where *B*_*c*_ is the parameter that represents the effective capillary pressure, and *S*_*e*_ is computed as follows


$$S_{e}=\;\frac{s_{w}-\;s_{wr}}{1-\;s_{or}-\;s_{wr}}$$ (28)

Employing nanofluids as a substitute for water represents a modern approach to enhancing flood efficiency. Adding nanoparticles to a base fluid, such as water, can elevate both its density and energy, resulting in an overall increase in fluid potency. Consequently, a larger volume of oil is extracted from reservoirs.


$$\frac{\partial \varphi S_{w}\psi_{w}}{\partial t}+\;u_{w}.\frac{\partial \psi_{w}}{\partial z}=\;\frac{\partial }{\partial z}.\left(\varphi S_{w}\psi_{w}D_{w}\frac{\partial \psi_{w}}{\partial z}\right)-R_{\iota }$$ (29)

The term *R*_*i*_ can be derived by using Eq. (30)


$$R_{i}\;=\;\frac{\partial \Omega }{\partial t}+\;\frac{\partial \Omega^{*}}{\partial t}$$ (30)

$$\frac{\partial \Omega }{\partial t}=\;K_{d}o\psi \;,\;\frac{\partial \Omega^{*}}{\partial t}\;=\;K_{p}o\psi \;$$ (31)

### 2.3 Boundary formulations

Using Eqs. (32)–(39), we can figure out the boundary conditions for MHD 3D prisms.

For *t* = 0, original saturation of water = 0, i.e.,


$$\;s_{w}^{0}=\;0,\;0\leq z\leq h$$ (32)

−n.ρu=0 (33)

−n.q=0 (34)

$$\rho u=\left({s_{w}\rho }_{w}+\;s_{o}\rho_{o}\right)U$$ (35)

$$-n.D_{C}\nabla c_{w}=0$$ (36)

At


$$\;t\;=\;0,\;s_{w}=0.15$$ (37)

At


$$\;t\;=\;0,\;\left\{ \;\;\;\;\;\begin{array}{c} \psi =0\\ \;\psi =\;\psi_{i} \end{array}\right.\;$$ (38)

At


$$t\;=\;0,\;\left\{ \;\;\;\;\begin{array}{c} \Omega =0\\ \;\Omega_{i}=\;0 \end{array}\right.\;$$ (39)

### 2.4. Analysis of mesh

**Fig 3:**
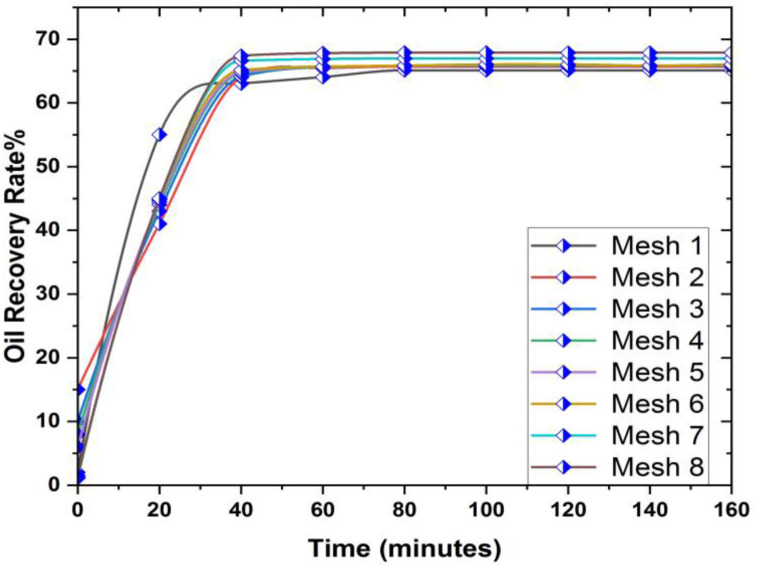
Results of Mesh in oil recovery.

Examining the mesh to select the optimal configuration for simulation is of utmost importance. In this paper, we conducted multiple experiments to determine the most suitable grid. The oil recovery achieved with varying mesh sizes is depicted in Fig 3, illustrating the impact of different grid sizes on oil recovery in the hexagonal prism.

**Fig 4:**
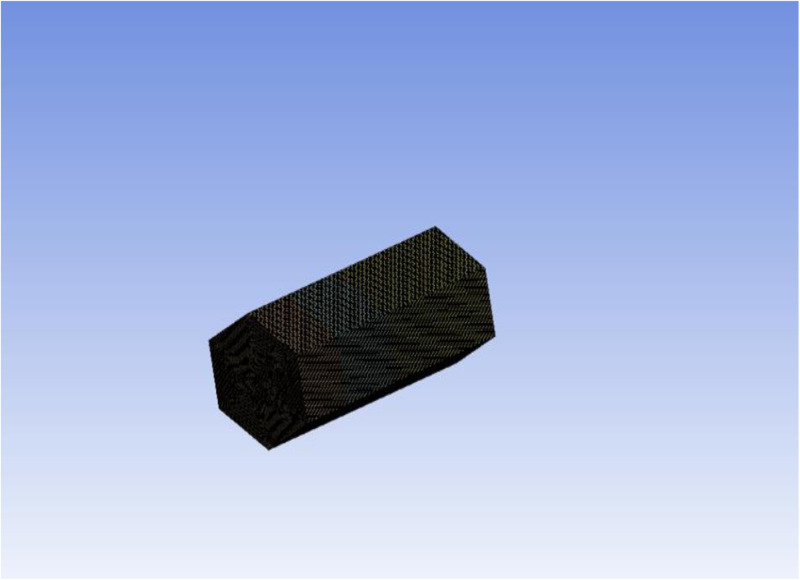
Contour representation of mesh analysis of 3D prism.

From Fig 3 it is observed that the oil recovery rates obtained from grids 6, 7 and 8 are identical and we choose mesh 6 for further investigation of our problem and the mesh diagram of the prism geometry is also presented in Fig 4).

Although Mesh 8 produced marginally more refined contours, the overall oil recovery difference compared to Mesh 6 was below 0.5%. As shown in Fig 3(a-b), both meshes reached similar saturation and pressure fields. Mesh 6 was selected for final simulations due to a 35% reduction in computation time without compromising accuracy.

### 2.5. Validation of the model

**Fig 5:**
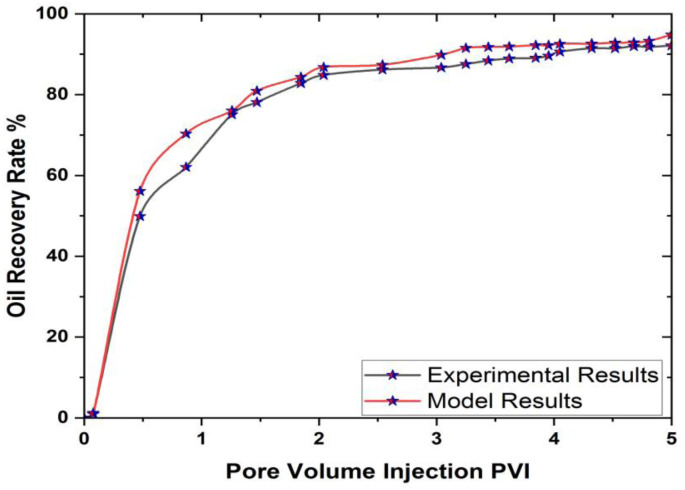
Validations of computational results with experimental work.

We validated our model with the experimental work published in [47]. It is important to note that for validation purposes, we neglected the magnetic field term in the model and compared it directly with the experimental data. It is evident that the results obtained from the considered hexagonal prism model closely match the experimental findings. For clarity, a graphical comparison is presented in Fig 5.

The oil recovery shown in Fig 5 corresponds to the base-case mesh sensitivity simulation (with default nanofluid and without optimized magnetic field settings). Final recovery values reported in validation studies reflect full-scale enhanced simulation conditions.

## 3. Results and discussion

In this section, we will provide comprehensive explanations of the results obtained from the MHD mathematical model for reservoir simulations. In the saturation contour plots, red regions represent zones of high residual oil saturation (i.e., lower recovery), while blue zones indicate effective oil displacement due to nanofluid injection.

### 3.1. Impact of the porosity

Porosity is important in enhanced oil recovery (EOR), especially when using nanofluids. Enhanced oil recovery is about getting more oil out of the ground than usual. Nanofluids are special because they have tiny particles in them that help in this process. Permeability affects how fluids and nanoparticles move in the ground, as well as how they are distributed and retained. Understanding how porosity changes and its impact on nanofluid behavior helps engineers make better plans to extract more oil from the reservoirs.

**Fig 6:**
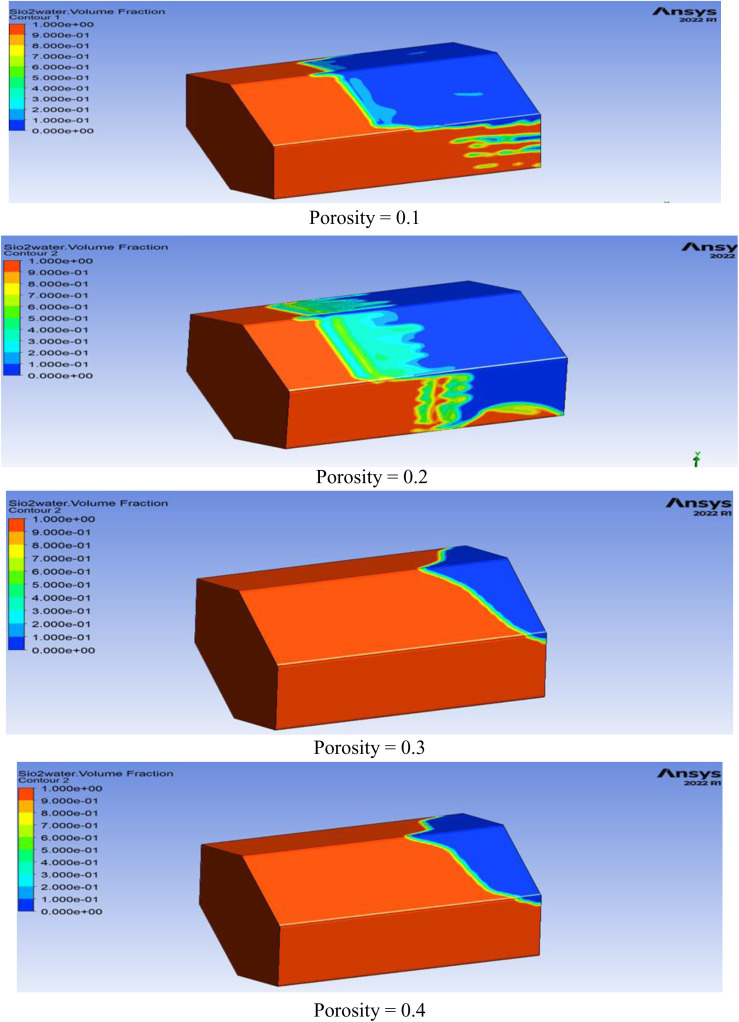
Oil saturation distribution after nanofluid injection. Red indicates high residual oil saturation (lower recovery), while blue indicates effective oil displacement (higher recovery).

The role of porosity to obtain optimal oil recovery in 3D hexagonal prism geometry at four different parameters, i.e., at φ = 0.1,0.2,0.3,0.4 and observed that as porosity value increases the oil recovery increases. The impact of this factor is shown in Fig 6.

From Fig 6, it is observed that as we increase the porosity parameter the oil recovery increases in 3D prism geometry, but optimal recovery is obtained at φ = 0.3 after this the oil recovery does not increase further. The reason behind the increase in the oil recovery with the increment of porosity parameter is that provides more pathways for fluid flow in the reservoir rock, facilitating the movement of injected fluids like nanofluids. This enhances contact between the injected fluids and trapped oil, increasing oil displacement and recovery efficiency.

**Fig 7:**
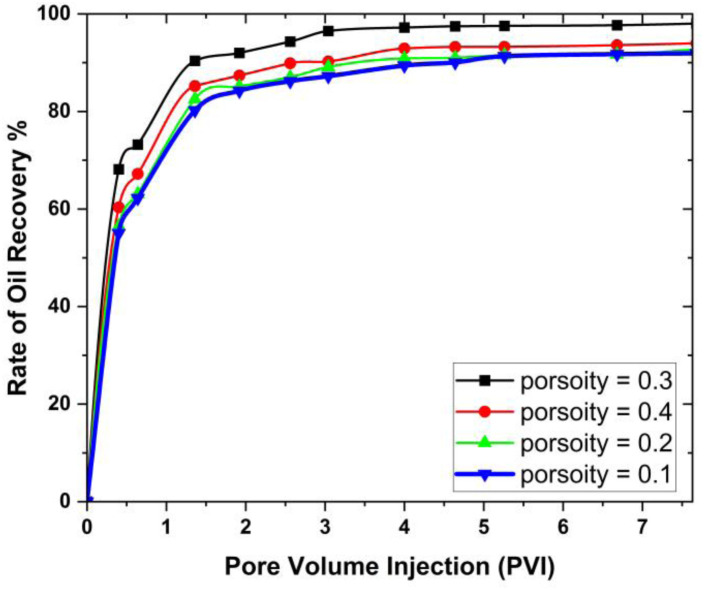
Graphical comparison for different parameters of porosity for oil recovery in MHD hexagonal prism.

The graphical comparison of the oil recovery at different parameters on 3D hexagonal prism is shown in Fig 7.

From Fig 7, it is clearly indicated that maximum oil recovery obtained from porosity 0.3 is 98.03% and minimum oil recover is at 0.1 which is 91.9% from 3D prism geometry. While surfactants effectively reduce interfacial tension (IFT) between oil and water, we recognize that additional methods, such as water injection, may be necessary to facilitate the movement of detached oil toward production wells. In conclusion, oil reservoirs frequently exhibit heterogeneity, implying they contain diverse rock types and characteristics throughout different areas. Porosity may vary significantly within a reservoir, impacting the patterns of fluid flow and the dispersion of injected nanofluids. Grasping the distribution of porosity is crucial for designing effective EOR approaches employing nanofluids.

### 3.2. Impact of the mass flow rate

**Fig 8:**
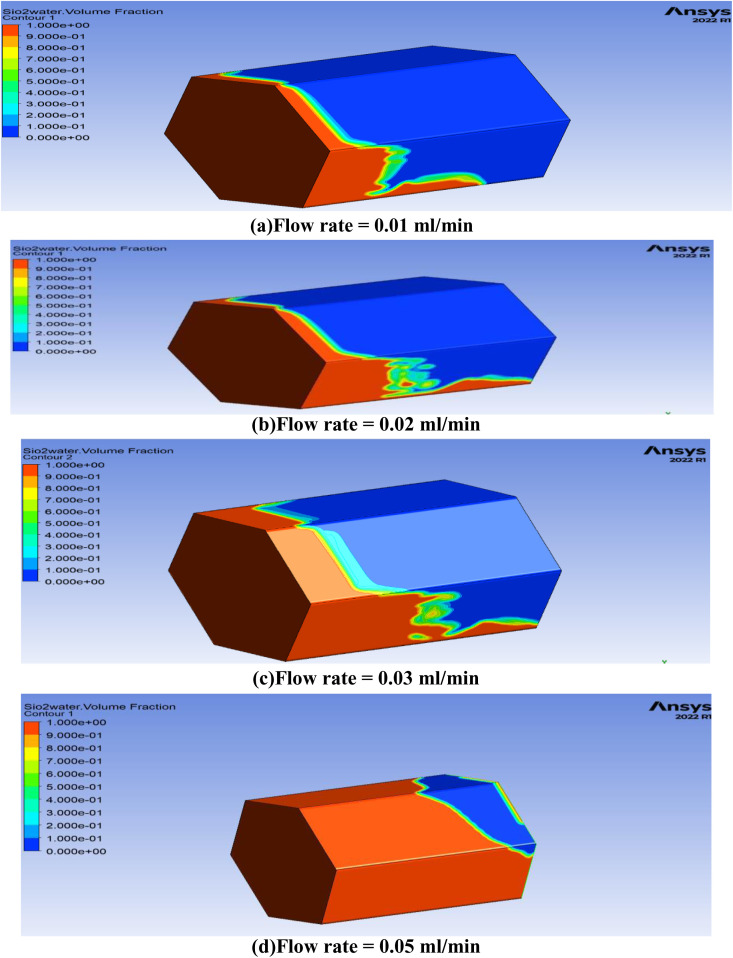
Comparison for flow rates of oil recovery in term of their contours. (Oil saturation distribution after nanofluid injection. Red indicates high residual oil saturation (lower recovery), while blue indicates effective oil displacement (higher recovery).

The rate at which mass flows is crucial in EOR procedures as it directly affects the efficiency of fluid displacement within the reservoir. Maximizing the mass flow rate enables the maximum oil recovery from the reservoirs. The parametric analysis of different values of the mass flow rate in 3D hexagonal prism to investigate the optimal value of the oil recovery factor is studied and the oil recovery obtained from these parameters is shown in Fig 8. Among the tested values, the flow rate of 0.05 mL/min was found to yield optimal oil recovery in the hexagonal prism model.

**Fig 9:**
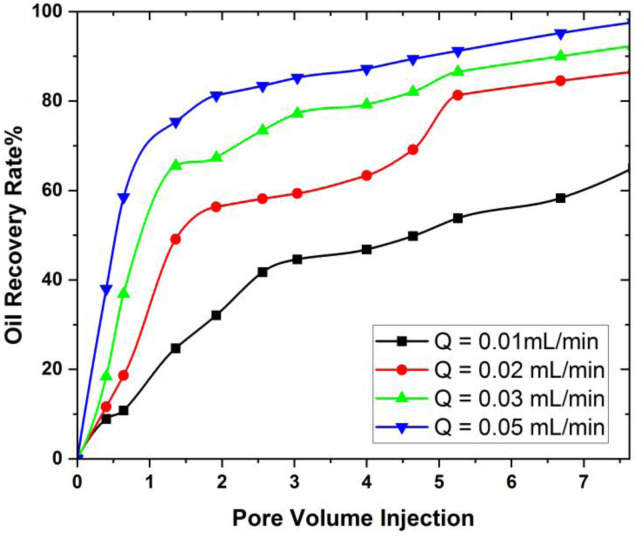
Relationship between the injected pore volume at various flow rates and the oil recovery factor. The results illustrate how increasing the injection rate influences the recovery trend across multiple pore volumes.

It is noticed from Fig 8, that oil recovery increases as flow rate decreases. When the flow rate reduce in oil recovery processes, it affords an extended duration for the interaction between injected fluids and the reservoir rock, facilitating superior fluid-rock interplay. This prolonged contact period bolsters mechanisms such as heightened sweep efficiency, mitigated viscous fingering, augmented capillary forces, minimized channeling, and overall enhanced blending between injected fluids and oil. These combined factors culminate in a more efficient displacement of oil from the reservoir, consequently leading to elevated recovery rates. The graphical explanation of these parameter on oil recovery rate is also shown in Fig 9.

It is also observed form Fig 9, that the lowest oil recovery rate obtained from 3D hexagonal prism is 60% at the flow rate of 0.01 mL/min, while the optimal value of oil recovery rate can be found at the flow rate of 0.05 mL/min. Contrary to some conventional expectations, the simulation results show that oil recovery increases with higher injection flow rates. Specifically, the maximum recovery factor was observed at 0.05 mL/min, indicating that enhanced nanofluid mobility and front propagation at this rate contributed to better sweep efficiency. Nevertheless, it’s important to acknowledge that the ideal flow rate for maximizing oil recovery is contingent upon a multitude of factors, encompassing reservoir characteristics, fluid properties, and the recovery technique being utilized. It is also observed that the maximum oil recovery is obtained at 0.05mL/min The analysis revealed that oil displacement becomes notably inefficient at flow rates below 0.03 mL/min. This threshold can be considered the critical flow rate for effective oil recovery under the modeled reservoir conditions.

### 3.3. Influence of nanoparticle volume fraction

**Fig 10:**
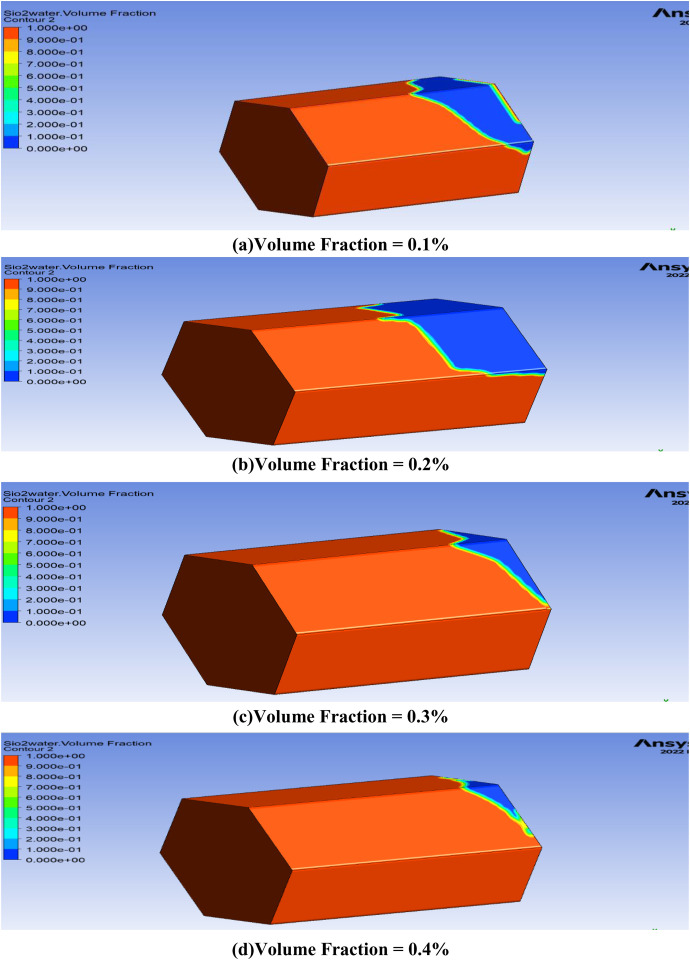
Contour representation on the effect of volume fraction on oil recovery in the reservoir geometry. (Oil saturation distribution after nanofluid injection. Red indicates high residual oil saturation (lower recovery), while blue indicates effective oil displacement (higher recovery).

Nanofluids enhance oil displacement primarily through three mechanisms: (i) increasing the viscosity of the displacing phase, thereby improving mobility control; (ii) reducing the interfacial tension (IFT) between oil and water, which facilitates the release of trapped oil droplets; and (iii) altering the wettability of the reservoir rock from oil-wet to water-wet, thereby enhancing spontaneous imbibition. These effects collectively lead to improved sweep efficiency and higher oil recovery. The concentration of nanoparticles is essential in oil recovery procedures because they have the capability to change the characteristics of the injected fluids and how they interact with the reservoir rock. When nanoparticles are added to the injected fluids, they have the capacity to adjust the viscosity, interfacial tension, and wettability of the fluid, all of which are critical aspects affecting oil recovery. The study investigates the nanoparticles volume fraction influence to ascertain the potential for extracting a larger amount of oil from the cavity. In this study, the term ‘concentration’ is used interchangeably with ‘volume fraction (), which represents the volumetric proportion of nanoparticles in the base fluid. Furthermore, the effect of the volume fraction of nanoparticles on oil recovery in hexagonal prism cavities is elucidated in Fig 10.

**Fig 11:**
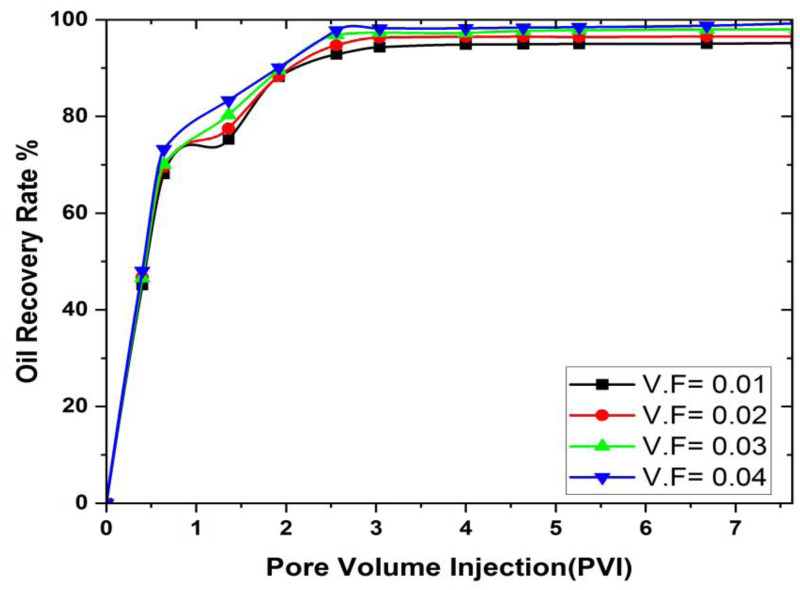
Rate of oil recovery at different values of volume fractions in MHD hexagonal prism.

Although Fig 10 does not directly depict IFT reduction, the improved oil recovery trends observed at higher nanoparticle concentrations are consistent with the known effects of IFT reduction, as supported in previous experimental studies. The graphical comparison of the oil recovery at different volume fractions is shown in Fig 11.

In Fig 11 the effect of the nanoparticles volume fraction on the oil recovery rate is positive for all pore volumes of SiO_2_. At Ψ = 0.01, the initial oil recovery is 43.38% at a pore volume of 0.4. This gradually goes up until the final pore volume, where the highest oil recovery is 94.98%. This shows that at value of Ψ = 0.01 the oil recovery provides a good effect on oil recovery. In the second parameter of volume fraction, at Ψ = 0.02, the maximum oil recovery reaches 95.89% of the final pore volume, which is 0.91% higher than at Ψ = 0.01. At Ψ = 0.03, the maximum oil recovery increases to 97.15% at the last pore volume injection, reflecting a rise of 2.17% compared to Ψ = 0.01 and an increase of 1.26% over Ψ = 0.02. Finally, at Ψ = 0.04, the maximum oil extracted from pore volume 7.68 is 99.01%, which is 4.13% higher than at Ψ = 0.01, 3.12% greater than at Ψ = 0.02, and 1.89% higher than at Ψ = 0.03. The improved oil recovery can be primarily attributed to interfacial tension reduction and wettability alteration caused by SiO₂ nanoparticles. These mechanisms enhance the displacing efficiency of the nanofluid. Viscosity effects are considered negligible at the tested concentrations The main reason is that nanoparticles can elevate the viscosity of the injected fluid, thereby enhancing sweep efficiency by reduce the mobility ratio between the injected fluid and the oil. This ensures that the injected fluid can displace a greater volume of oil within the reservoir, ultimately maximizing oil recovery.

### 3.4. Impact of magnetic field

**Fig 12:**
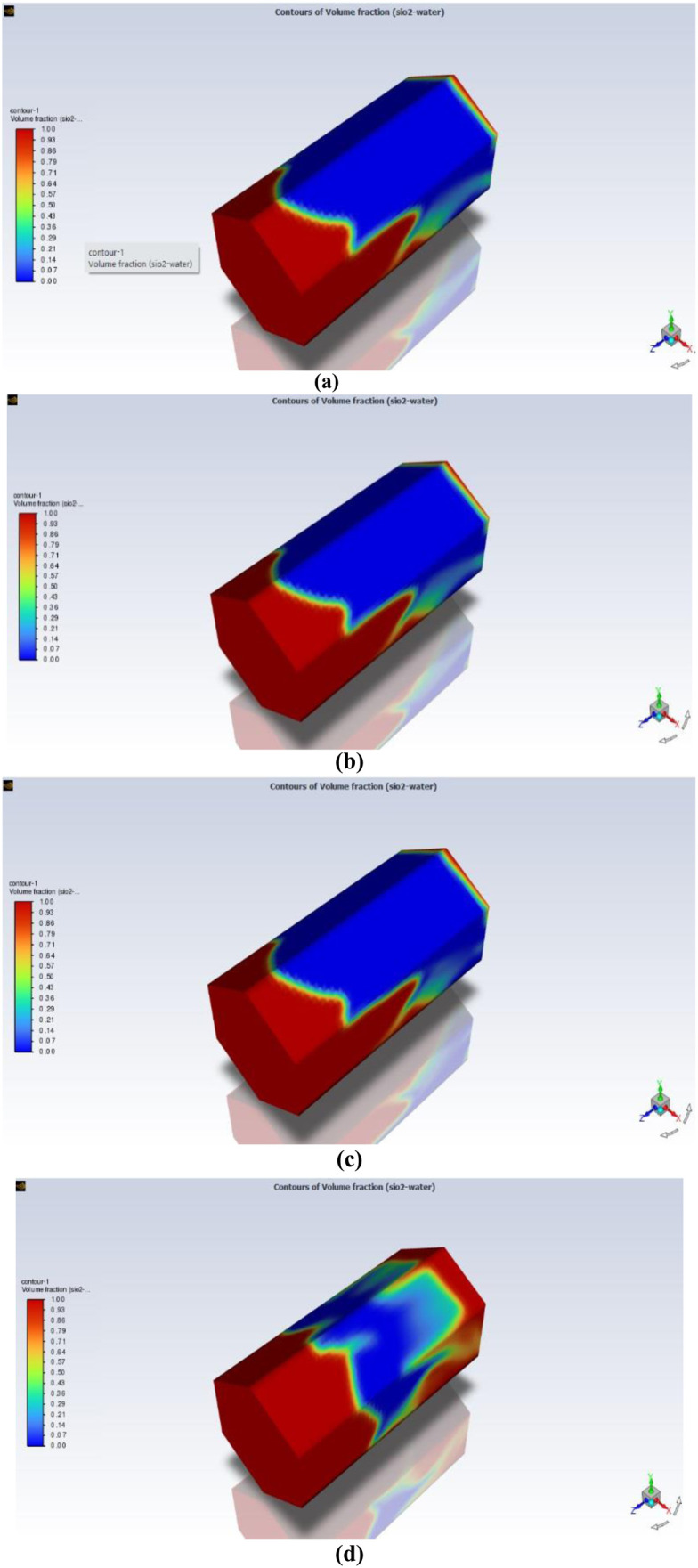
Cantor comparison of oil recovery in MHD hexagonal prism. a) z =  0, b) z = 0.05m, c) z  = 0.2 m and d) z = 0.1 m. (Oil saturation distribution after nanofluid injection. Red indicates high residual oil saturation (lower recovery), while blue indicates effective oil displacement (higher recovery).

The effect of magnetic field on 3D prism to obtain oil recovery at different pore volumes and different distances is explained in this section. In the simulation setup, a uniform magnetic field was applied externally from the left side of the cavity, acting perpendicularly to the main flow direction. The field was tested at four locations z = 0, 0.05, 0.1, and 0.2 meters to evaluate its influence on oil displacement within the reservoir. Fig 12 provides a visual and cantor comparison of oil recovery at various magnetic field places. In the simulations, a static magnetic field of intensity Br = 0.25 T. was applied externally from the left boundary of the prism, perpendicular to the direction of fluid flow (along the z-axis). The spatial variation of the magnetic field with distance was modeled using a hyperbolic tangent profile to simulate realistic decay.

From Fig 12, it is evident that oil recovery increases as the distance between the cavity and the magnetic field decreases. This phenomenon occurs because the presence of a magnetic field increases the viscosity of the injected fluid, leading to enhanced fluid flow within the cavity. Consequently, flow resistance decreases, and fluid mobility improves, allowing the injected fluid to displace more oil from the cavity. Therefore, to achieve maximum oil recovery, it is advisable to minimize the distance between the magnetic field and the reservoir geometry.

**Fig 13:**
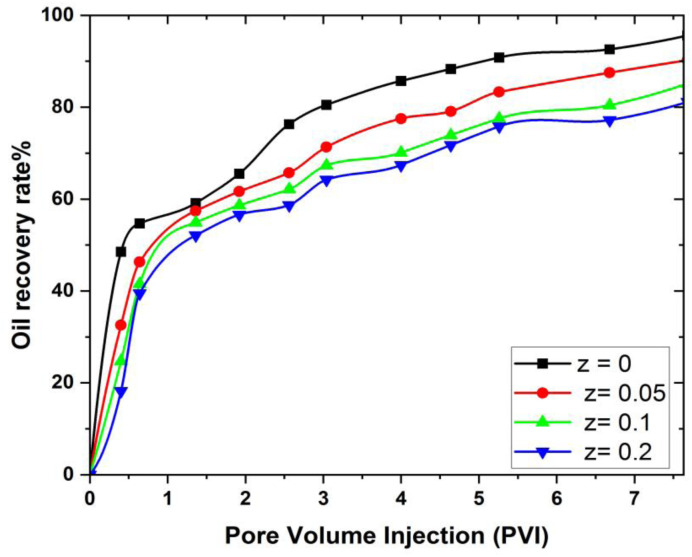
Graphically comparison of oil recovery in MHD hexagonal prism in the presence of external magnetic field.

From Fig 13 when z = 0, the oil recovery is only 48.52% when the pore volume is 0.4, but this number steadily increases until it reaches 95.58% when the pore volume is 7.68. At z = 0.05 m, the oil recovery at the first pore volume is 32.54%, which is 15.98% lower than z = 0, and at the last pore volume, the maximum amount of oil extracted is 90.21%, which is 5.37% lower than z = 0. On the other hand, at z = 0.05 m, the oil recovery at the last pore volume is 90.21%, which is 5.37% lower than z = 0. At z = 0.1 m, the amount of oil recovered at the initial pore volume is 7.8%, which is 23.78% less than the amount that we get from z = 0.1 and z = 0 meter distances, and also at the last pore volume, the final oil recovery is 85.12%, which is also 5% and 10.46 less than the oil that was recovered from z = 0.05 and z = 0 meter distances, respectively. The amount of oil recovery attained at distance z = 0.2 m is 14.13%, 9.08%, and 3.99% lower as compared to the distance from the magnetic source of the cavity at z = 0, 0.05, and 0.1 m, respectively. It means that the oil recovery rate decreases if the distance of the magnet increases.

### 3.5. Comparison of oil recovery

**Fig 14:**
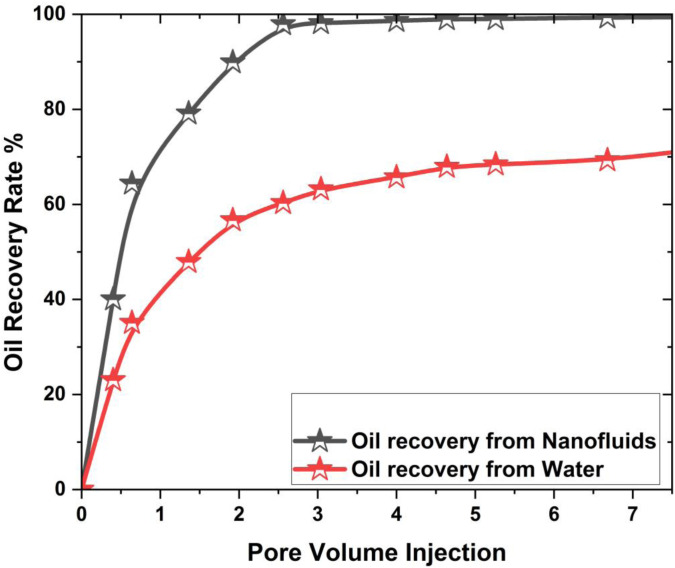
Comparison of oil recovery from nano flooding vs water flooding in the existence of the magnetic field.

In this section the compassion of nanoflooding with respect to the waterflooding is provided. To ensure comparability, the baseline water flooding simulation was performed under the same reservoir and operational conditions as the nanofluid (SiO₂) flooding case. This includes identical injection flow rate (0.05 mL/min), injection pressure (1.2 atm), and total injected pore volume (1 PV).The oil recovery obtained from magnetic nanoparticles in 3D hexagonal prism with water in the presence of nonmagnetic field provided and their comparisons graphs are shown in Fig 14. The nanofluid flooding results presented here, used for comparison with conventional water flooding, are taken from Section 3.3, where a nanoparticle volume fraction of Ψ = 0.04 was used. This concentration yielded the highest oil recovery in the concentration sensitivity analysis

From Fig 14, it is clearly observed that the nanoflooding provides higher oil recovery as compared to the water flooding in every case. It is also noted that the oil recovery for magnetic nanoparticles is 29.08% as compared to the water flooding. The reason to increase in oil recovery by using nanofluid is that nanoparticles increase fluid viscosity, improving mobility control for more effective oil displacement. When nanoparticles are added to the injected fluid, it becomes thicker. This makes it easier to control how the fluid moves and makes it easier to move oil within the tank. These nanoparticles also change how wet the rock is, which lets oil out of the holes and gives the injected fluid and oil more space to touch. Also, nanofluids improve the efficiency of the sweep by allowing entry to parts of the reservoir that haven’t been recovered before and by reducing water fingering through selective plugging. Also, nanoparticles cause an effect called “disjoining pressure,” which reduces capillary forces and frees more oil. They also reduce the tension between oil and water, which makes it easier to move the oil.

## 4. Conclusion

This research explores the impact of 3D complex prism geometry on enhanced oil recovery (EOR) in hypothetical oil unconventional reservoirs under the presence of the external magnetic field placed at different positions to find out optimal oil recovery, such an area was not extensively studied in the existing literature. Based on the results there are the following conclusion remarks:

i Maximum oil recovery obtained from porosity 0.3 is 98.03% and minimum oil recover is at 0.1 which is 91.9% from 3D prism geometry.
ii The lowest oil recovery rate obtained from 3D hexagonal prism is 60% at the flow rate of 0.01 mL/min, while the optimal value of oil recovery rate can be found at the flow rate of 0.05 mL/min. Nevertheless, it’s important to acknowledge that the ideal flow rate for maximizing oil recovery is contingent upon a multitude of factors, encompassing reservoir characteristics, fluid properties, and the recovery technique being utilized.
iii The effect of the nanoparticle volume fraction on the oil recovery rate is positive for all pore volumes of SiO_2_
iv Oil recovery increases as the distance between the cavity and the magnetic field decreases.

In future this mathematical model can be extended for more complex geometries in the existence of higher temperate and higher pressure to obtain maximum oil recovery rate.

### Nomenclature

**Table:** 

$a_{1},\;b_{1}$	Magnetic Parameters
$B_{r}$	Residual Magnetization
$B_{c}$	Effective Capillary Pressure
c	Capillary
F	External Magnetic Force
$f_{w}$	Flow Fraction of Water
$f_{o}$	Flow Fraction of Oil
g	Gravity
H	Magnetic Field Strength
k	Thermal Conductivity
$K_{r}$	Relative Permeability
K	Permeability
l	Distance between magnetic poles
p	Pressure
$p_{c}$	Capillary Pressure
$p_{w}$	Water Pressure
$p_{m}$	Magnetic Pressure
$p_{s}$	Magnetostriction Pressure
$p_{n}$	Magnetic Normal Pressure
R	Loss of Nanoparticles
s	Saturation
$s_{w}$	Water Saturation
$s_{o}$	Oil Saturation
$s_{e}$	Effective Saturation
U	Fluid Velocity
V	Specific Volume
**Greek Letters**	
ρ	Density
μ	Viscosity
Φ	Porosity
$\gamma_{o}$	Mobility ratio of Oil
$\gamma_{w}$	Mobility ratio of Water
Ψ	Nanoparticle Fraction of Concentration
